# Application of the Response Surface Methodology to Optimize the Fermentation Parameters for Enhanced Docosahexaenoic Acid (DHA) Production by *Thraustochytrium* sp. ATCC 26185

**DOI:** 10.3390/molecules23040974

**Published:** 2018-04-22

**Authors:** Kang Wu, Lijian Ding, Peng Zhu, Shuang Li, Shan He

**Affiliations:** 1 Li Dak Sum Yip Yio Chin Kenneth Li Marine Biopharmaceutical Research Center, Ningbo University, Ningbo 315211, China; wukang3@163.com (K.W.); zhupeng@nbu.edu.cn (P.Z.); 2 Key Laboratory of Marine Biotechnology of Zhejiang Province, Ningbo University, Ningbo 315211, China; 18857497140@163.com; 3 Ningbo Entry-Exit Inspection and Quarantine Bureau Technology Center, Ningbo 315211, China

**Keywords:** *Thraustochytrium*, docosahexaenoic acid (DHA), Plackett-Burman designs, response surface methodology

## Abstract

The aim of this study was to determine the cumulative effect of fermentation parameters and enhance the production of docosahexaenoic acid (DHA) by *Thraustochytrium* sp. ATCC 26185 using response surface methodology (RSM). Among the eight variables screened for effects of fermentation parameters on DHA production by Plackett-Burman design (PBD), the initial pH, inoculum volume, and fermentation volume were found to be most significant. The Box-Behnken design was applied to derive a statistical model for optimizing these three fermentation parameters for DHA production. The optimal parameters for maximum DHA production were initial pH: 6.89, inoculum volume: 4.16%, and fermentation volume: 140.47 mL, respectively. The maximum yield of DHA production was 1.68 g/L, which was in agreement with predicted values. An increase in DHA production was achieved by optimizing the initial pH, fermentation, and inoculum volume parameters. This optimization strategy led to a significant increase in the amount of DHA produced, from 1.16 g/L to 1.68 g/L. *Thraustochytrium* sp. ATCC 26185 is a promising resource for microbial DHA production due to the high-level yield of DHA that it produces, and the capacity for large-scale fermentation of this organism.

## 1. Introduction

Docosahexaenoic acid (DHA) plays a significant role as a nutrient required by both invertebrates and vertebrates. Since DHA is indispensable for prenatal and postnatal development of visual and other perceptive functions, this compound is added to many infant dietary formulas [1,2]. Additionally, it has been observed that DHA is correlated with enhanced quality of life and reduced risk of cardiovascular and neurological diseases [3]. 

Mammals lack the enzymes required to produce DHA de novo, so this substance must be obtained through the diet. A variety of marine fish species are currently the primary commercial sources of DHA. However, fish-derived DHA is associated with many shortcomings, including unstable quality, a fishy odor, environmental pollution, high processing costs and a tendency to oxidize [4,5]. Accordingly, efforts have been made to divert commercial DHA production towards the exploitation of oleaginous microorganisms that biosynthesize DHA, such as thraustochytrids and marine diatoms [6]. *Thraustochytrium*, unicellular eukaryotic marine protists, have been isolated mainly from coastal marine environments [7]. *Thraustochytrium* strains have attracted attention from researchers worldwide, since these can build dense biomass in culture and produce polyunsaturated fatty acids (PUFAs), especially those from the omega−3 series, such as DHA [8]. Therefore, some *Thraustochytrium* strains are considered to be prospective alternatives to marine fish oils as new sources of DHA for use in aquaculture products and food additives.

To optimize DHA production, several chemical parameters, including carbon and nitrogen sources at different concentrations, as well as physical parameters, including initial pH and inoculum age, must be considered [9]. It was observed that thraustochytrids isolated from different environments also have variant DHA production levels [10,11]. The fermentation conditions must, furthermore, have a decisive role in regulating the DHA production of thraustochytrids [12]. With the aim of high DHA productivity, a temperature shift regime resulted in a DHA content of 52% of total fatty acids. A decline in temperature from 30 °C to 20 °C yielded high levels of DHA, making 30 °C the optimal culture temperature for the highest biomass growth [13]. Glucose and glycerol are used as a mixed carbon source for *Aurantiochytrium limacinum* SR21, and DHA productivity was 15.24% higher than that obtained using glucose as a single carbon source [14]. The continuous culture method was carried out to feed *A. limacinum* SR21, whereby the yield of biomass was 11.78 g/L and DHA concentration reached 1.74 g/L [15]. Response surface methodology (RSM) is known to be effective for the optimization of multiple factors for fermentation yields [16]. The empirical model developed by RSM was adequate to ascertain the possible interactions between the studied parameters and the response of DHA production. For example, RSM was previously used in optimizing significant fermentation variables (glucose concentration, yeast extract addition, NaCl content, pH, and incubation time), leading to a maximum DHA concentration of 516 mg/L by *Schizochytrium* sp. S31 [17]. 

This work attempts to optimize the fermentation parameters in the cultivation of *Thraustochytrium* sp. so that it may enhance the DHA yield. The influence of fermentation parameters on the production of DHA were investigated through the Box-Behnken design of experiments. 

## 2. Results

### 2.1. Optimization of Culture Conditions by RSM

**Figure 1:**
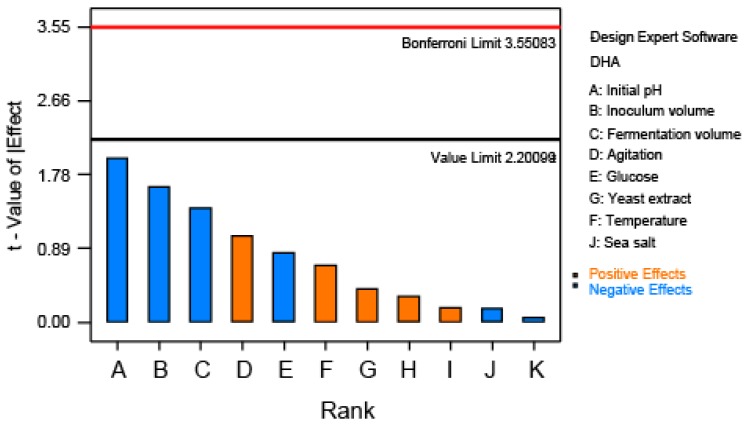
Pareto chart of each factor standard effects on DHA production.

Plackett-Burman design methods can be used in the screening of the key influential factors compared with other statistical designs on the experimental response. Eight variables were selected in the screening approach with Plackett-Burman experimental design, suggesting 12 trial runs. The production of DHA was selected as the observed response to determine the effects of the variables studied. Table 1 shows the experimental parameters investigated and the levels of each used in the experimental design, whereas the design matrix is shown in Table 2. Pareto analysis was used to determine the contribution of the screened variables (Figure 1), which showed that the initial pH had the highest positive effect on DHA production, followed by inoculum volume, and fermentation volume. These three factors were determined to be of the highest relative importance that influence DHA production compared with the other fermentation parameters tested and, hence, each was selected for further optimization.

### 2.2. Optimization of Fermentation Parameters by BBD

The most important parameters determined in this study by Plackett-Burman design experiments for the efficiency of DHA production were fermentation volume, inoculum volume, and initial pH. In order to study the combined effect of these factors, secondary experiments were performed with different combinations of the parameters. The range of values for the input variables is shown in Table 3. Trial runs were planned to obtain a quadratic model consisting of 12 trials plus center points. Thus with the aim of assessing the combined effect of these factors, 17 experiments were performed for different combinations of the parameters using statistically designed experiments. The design matrix of the parameters in coded units is listed in Table 4, along with the predicted and experimental values of response. The final regression equation in terms of coded factors was evaluated by Design-expert software, which yielded Equation (1) below:

(1) $$\begin{array}{c} Y_{DHA}=1.22-0.21X_{1}+0.071X_{2}+0.092X_{3}-0.046X_{1}X_{2}-0.099X_{1}X_{3}-0.036X_{2}X_{3}\\ +3.463\times 10^{-4}X_{1}^{2}+0.16X_{2}^{2}+0.14X_{3}^{2} \end{array}$$

In Equation (1), Y is the predicted response of DHA production, $X_{1}$, $X_{2}$, and $X_{3}$ are the coded values of the test variables, fermentation volume (mL), inoculum volume (%), and initial pH, respectively. The statistical significance of Equation (1) was checked by the *F*-test, and the analysis of variance (ANOVA) for response surface quadratic model is summarized in Table 5.

**Figure 2:**
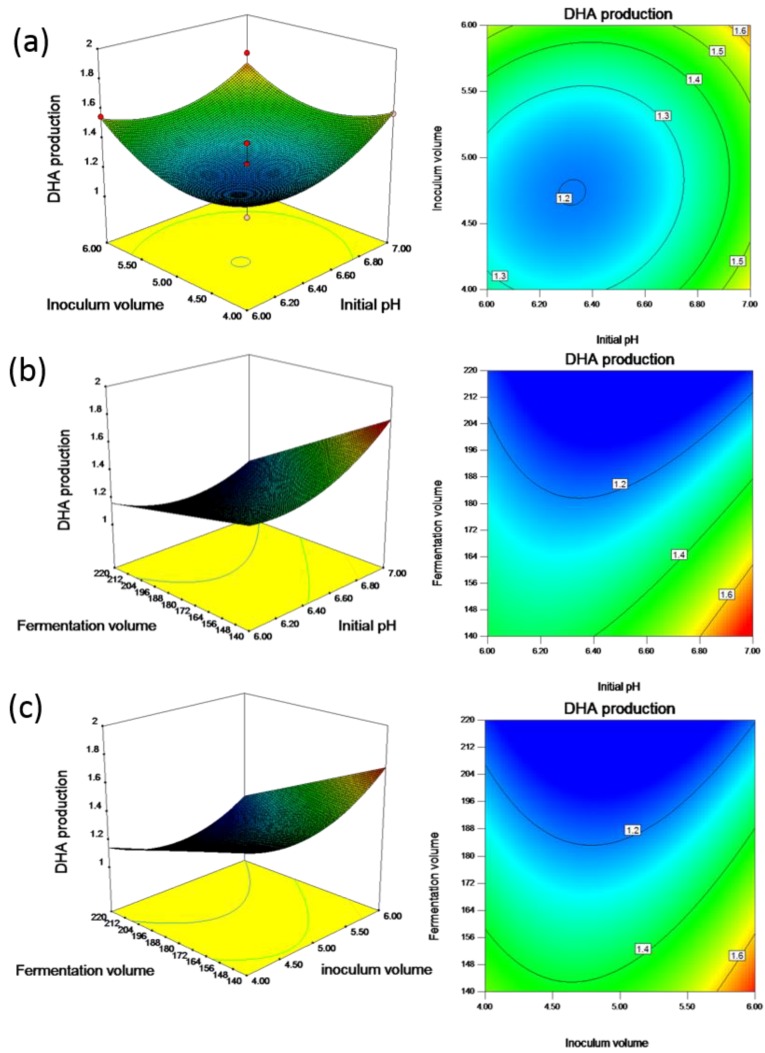
Response surfaces and corresponding contours of DHA production in terms of interaction between (**a**) inoculum volume (mL) and initial pH; (**b**) fermentation volume (mL) and initial pH; and (**c**) fermentation volume (mL) and inoculum volume (mL).

The three-dimensional response surface plots generated from BBD experiments are the graphical representations of the regression equation, and these plots are shown in Figure 2. The main goal of RSM is to efficiently track the optimum values of the process parameters such that the response is maximized. Software-based numerical optimization of the overall desirability function was carried out to find the optimal response within the specified ranges of the variables in the optimization of fermentation for DHA production. The predicted optimal values for the parameters were as follows: fermentation volume, $X_{1}=$ 140.47 mL, inoculation volume, $X_{2}=4.16\%$, and initial pH, $X_{3}=6.89$. To confirm the validity of predicted response, three successive experiments were conducted using the optimized parameters, and the statistical model and regression equation were validated and adequate. The average amount of biomass obtained in these experimental runs was 15.01 g/L. The obtained DHA production from the optimized condition was 1.68 g/L, near to the predicted result of 1.77 g/L.

## 3. Discussion

Response surface methodology is suitable for processes optimization for many industrial products, including paints and coatings, foods and beverages, and pharmaceuticals. Compared with one-factor-at-a-time experiments, statistically designed experiments are able to describe the effect of the interactions among the factors in linear and quadratic terms. In the present study, the optimization of DHA production by *Thraustochytrium* sp. ATCC 26185 was divided into two phases: a screening of main effects of the selected variables and a response optimization. Reduction in the initial number of variables was carried out through Plackett-Burman design and, among eight tested variables, the initial pH, inoculum volume, and fermentation volume were taken for BBD of RSM to assess their effects on DHA production by *Thraustochytrium* sp. ATCC 26185.

**Figure 3:**
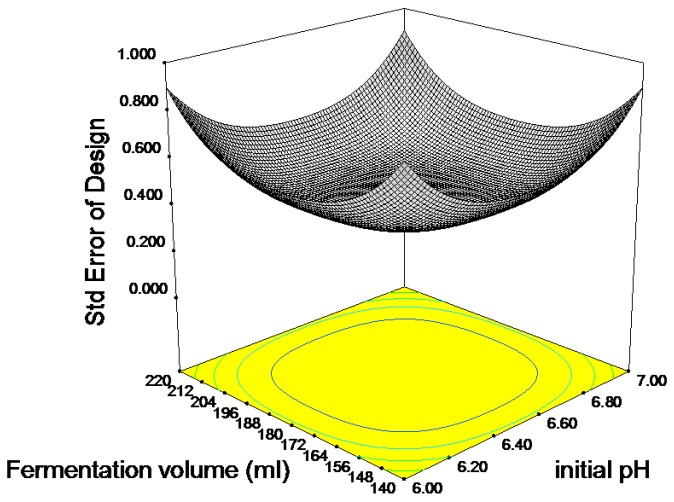
Three-dimensional standard error plot of the model holding initial pH and fermentation volume.

A large model *F*-value indicates that most of the variation in the response can be explained by a given regression equation. The ANOVA result for DHA production by *Thraustochytrium* sp. ATCC 26185 in this study showed an *F*-value of 9.93, which indicated that the terms in the model had a significant effect on the response. The probability *p*-value of 0.0031 for DHA production reinforced that the model terms are statistically significant at the 95% confidence level. The results of ANOVA also provide a term for residual error, which measures the amount of variation in the response data that was left unexplained by the model. In the present study, the *R*^2^ and Adj *R*^2^ values for DHA production were 0.9210 and 0.8559, respectively. It can, thus, be understood that at least 7% of the experimental results were not explained by the proposed model. The predicted *R*^2^ is the measure of the variation in data explained by the model. The “Pred *R*^2^” of 0.7290 was in reasonable agreement with the “Adj *R*^2^” of 0.8559. The adequate precision value is the signal to noise ratio, and a value >4 is desirable in support of the fitness of the model [18]. The ratio determined of 9.047 indicated an adequate signal, suggesting that these models can be used to navigate the design space (Figure 3).

The ratio of triacylglycerols to phospholipids, as well as the fatty acid profile of *Thraustochytrium* is known to depend on culture conditions [19]. The optimization of culture conditions (pH, dissolved oxygen, and temperature) led to a significant increase in the amount of eicosapentaenoic acid (EPA) produced by marine bacteria, where the amount of EPA increased from 33 mg/L to 350 mg/L [20]. Production of EPA comprises biomass producing and lipid generating stages. During the first phase, the biomass is increased by supplying carbon and nitrogen. In the second phase, carbon alone is supplied to enhance lipid levels. Accumulation of DHA in the form of triacylglycerols or neutral lipids is important for these protists. It was observed that neutral and polar lipids were produced in equal amounts in the early growth phase in *Schizochytrium* sp. strain SR21 and microalgae, but on further cell growth only the neutral lipids increased [21]. The DHA content among total fatty acids was only 8.7% in mature cells of *S*. *limacinum* SR21 compared to 62% in young ones, suggesting the transfer of the fatty acids of polar lipids to neutral lipids [22]. The biological significance of DHA in thraustochytrid cells was studied to show that DHA is conserved in the form of fatty acid energy reserves and utilized during starvation [23]. A decline in total lipids was observed with the extension in the starvation phase. Therefore, obtaining the maximum possible biomass with a high amount of total lipids in the shortest possible time is recommended for the strategy in development for DHA production. In this study, the inoculum volume was manipulated for biomass growth and lipid accumulation to reach the stationary phase. It was found that inoculum volume and initial pH interacted negatively, with a coincident decrease in both of these variables resulting in an increase in DHA production. As shown in Figure 2a, the pattern of the effect of inoculum volume on DHA can be visualized using a three-dimensional response surface plot and corresponding contour plot, and the production of DHA increased as the inoculum volume increased. The size of the inoculum obviously plays a significant role in the growth phase. This likely occurred because the increase in inoculum sizes manipulated microbial growth and lipid biosynthesis, whereas excessive inoculation led to the shortening of the fermentation cycle. The results also showed that an appropriate inoculum volume and initial pH enhanced the production of DHA.

The *p*-value of each variable shown in Table 5 show that the fermentation volume in flasks was the most significant growth factor affecting the growth and DHA production. The increase in the amount of liquid volume in the shake flask could cause the dissolved oxygen to decrease, which inhibits cell growth. It has been reported that the transformation of saturated fatty acids into unsaturated fatty acids occurred when oil-producing strains grow in oxygen-abundant environments [24]. The volume of the liquid medium was also noted that DO was affected, thus resulting in a lower DHA yield [25]. A large volume of liquid medium (more than 40 mL in a 250 mL flask) can also lead to a low DHA yield [16]. The effect of fermentation volume on DHA production may be partially due to the fact that the solubility of oxygen significantly increases at lower liquid volume, which may lead to a relatively higher production of DHA. The higher level of DHA observed at higher DO may be due to the enhanced biosynthesis of DHA as a response to a high DO concentration in the surrounding environment. It has been reported that a stepwise aeration control strategy was adopted for *Schizochytrium* sp., whereby biomass reached 71 g/L and total fatty acids reached 35.75 g/L [26]. It was also observed that a DHA concentration, DHA productivity, and conversion yield of 28.93 g/L, 301 mg/L/h, and 0.44 ± 0.02 g/g, respectively, by *Schizochytrium* sp. S31 under constantly high oxygen transfer conditions [27]. Thus, obtaining high amounts of DHA requires a higher level of DO.

Table 5 indicates that the interaction between the fermentation volume and initial pH has a significant effect on DHA production, whereas the quadratic effect of pH had a significant impact on the amount of biomass and DHA production. Figure 3b shows the three-dimensional response surface plot for this interaction. From this figure, it can be deduced that providing high levels of fermentation volume and low levels of pH has a negative effect on DHA. Thus, to obtain high amounts of DHA, either one of these factors must be kept at a low level while maintaining the other factor at a high level. The effect alters permeation of ions, acids and bases into the cell, and affects the biochemical metabolism. It was previously found that DHA formation was slightly affected by medium pH with the highest DHA proportion of total fatty acids at pH 7.2 in *Crypthecodinium cohnii*, whereas the saturated fatty acids proportion of total fatty acids at this pH value were the lowest [28]. The production of lipid and DHA by *Schizochytrium* sp. S31 incubated at initial pH levels of 5.0 and 6.0 was low. An initial pH of 8.0 for cultivation was too basic for growth and there was a high level of residual glucose, 16 g/L, in the medium after four days of cultivation [17].

To date, numerous studies have been conducted to enhance DHA production of *Thraustochytrium* using various modes of fermentation and strategic techniques. In an attempt to enhance DHA production by *Thraustochytrium* sp. ATCC 26185, Furlan et al. performed the conventional one-factor-at-a-time optimization, and the experiment showed the highest DHA yield (1.16 g/L) using different glucose and nitrogen concentrations [29,30]. In comparison, this isolate produced 1.68 g/L in the current study using the more sophisticated statistical design of experiments in shake-flask, which was 45% higher than prior to optimization. 

## 4. Materials and Methods 

### 4.1. Microorganisms and Culture Conditions

*Thraustochytrium* sp. ATCC 26185 was purchased from the American Type Culture Collection (Manassas, VA, USA). An extract produced from *Thraustochytrium* sp. ATCC 26185 was previously shown to contain high amounts of lipids, up to 32% of the biomass, and DHA comprised as much as 25% of the total fatty acid content [31]. This organism was maintained at room temperature on GYP agar plates containing 4 g/L glucose, 2 g/L yeast extract, 2 g/L peptone, 30 g/L artificial seawater salt, and 15 g/L agar. The single colonies from a plate culture were inoculated into 50 mL seeding broth (in 250 mL flask) containing 40 g/L glucose, 5 g/L yeast extract, 5 g/L peptone, and 30 g/L sea salt, then were grown at 28 °C on a shaker incubator with 200 rpm agitation.

### 4.2. Experimental Design: Plackett-Burman Design

Plackett-Burman design is applied to evaluate the relative importance of several fermentation parameters that influence the desired response, and enables one to screen N variables in at least N + 1 experiments [32]. To increase the response, each factor selected was tested in two levels, high (+1) and low (−1). A Plackett-Burman design of the experiments was formulated for eight factors using the Design-Expert version 8.0 software (Stat-Ease, Inc., Minneapolis, MN, USA). For the present study, the selected factors included the levels of glucose, yeast extract and sea salt, fermentation volume, inoculum volume (mid-exponential phase), temperature, initial pH, and agitation rate. The levels of each factor were determined based on prior experience. The effect of each variable was estimated from by Equation (2), as follows:

(2) $$E\left(xi\right)=\frac{\sum^{\text{​}}M_{i}^{+}-\sum^{\text{​}}M_{i}^{-}}{N}$$

where $\sum^{\text{​}}M_{i}^{+}$ and $\sum^{\text{​}}M_{i}^{-}$ are the sum of the responses for which variable Xi is in the high level (Xi = +1) and the low level (Xi = −1), respectively. In this equation, N is the total number of trials conducted. 

Each trial run was performed in triplicate, and the average of DHA production from *Thraustochytrium* sp. ATCC 26185 was taken as the response. Pareto chart analysis was used to evaluate the variables, which presents the impact of each variable on DHA production, and the most significant variables were further investigated by Box-Behnken design of response surface methodology.

### 4.3. Experimental Design: Box-Behnken Design

It is possible to derive an expression for an overall performance measure based on the response values obtained from experiments at particular combinations of input variables [33]. Box-Behnken design (BBD) was performed to ascertain the main effects of the parameters studied, the interactions among them and the quadratic effect for the responses. A three-level, three-factorial BBD system was used to investigate and validate the fermentation parameters affecting production of DHA by *Thraustochytrium* sp. ATCC 26185. To evaluate the influence of significant factors, three independent variables were chosen after Pareto analysis of the Plackett-Burman experimental results: fermentation volume, inoculum volume and initial pH. The ranges of variables were assigned based on path of steepest ascent [34]. A total of 17 experiments were conducted based on the output of Design Expert software.

The numbers of experiments were calculated as N in Equation (3), as follows:

(3) $$N=k^{2}+k+n_{0}$$

In Equation (3), k is the variable number and $n_{0}$ is the number of the center point. Quadratic and interaction effects of the independent variables were evaluated for RSM. The sequential *F*-test, lack-of-fit test and other adequacy measures were used in selecting the best model [35]. The relationship between the response and the input process parameters were performed by using Equation (4):

(4) $$Y=ƒ\left(x_{1}+x_{2},\ldots ,x_{n}\right)+\varepsilon$$

In Equation (4), ƒ is the real response function its format being unknown, and ε is the residual error which describes the differentiation that can be included by the function ƒ. In consideration of the linear terms, square terms and linear interaction items, the second order equation response was modeled by Equation (5) to correlate the dependent and independent variables, as follows:

(5) $$Y=\beta_{0}+\sum^{\text{​}}\beta_{i}x_{i}+\sum^{\text{​}}\beta_{ii}x_{ii}^{2}+\sum^{\text{​}}\beta_{ij}x_{i}x_{j}+\varepsilon$$

In Equation (5), Y is the predicted response, $\beta_{0}$ is the model constant, $\beta_{i}$ is the regression coefficient for the input factor $x_{i}$, $\;\beta_{ii}$ is the regression coefficient for interactions, and $\beta_{ij}$ is the regression coefficient for square effects between the input factor $x_{i}$ and $x_{j}$. The optimum values of the selected variables were determined by solving the regression equation and analyzing the three-dimensional surface plots. A fermentation experiment was employed to evaluate the DHA production of *Thraustochytrium* sp. ATCC 26185 using the predicted optimum parameter values.

### 4.4. Fatty Acid Methyl Ester Extraction, Preparation, and Analysis

Dried cells (20 mg) were suspended in 1.5 mL methanol/acetyl chloride (10:1, *v*/*v*), 1 mL of hexane and 15 μg nonadecanoic acid were added and heated at 70 °C for 2 h in sealed tubes. After cooling, 2.5 mL 6% K_2_CO_3_ and 1 mL hexane were then added to the extracts, the mixture shaken, centrifuged, and the upper layer separated.

The profiling of extracted FAMEs was performed by GC-MS with a SPB-50 fused silica capillary column, 30 m × 0.25 mm × 0.25 μm (Supelo, Bellefonte, PA, USA) installed in a QP2010 GC equipped with an AOC-20 autosampler (Shimadzu, Kyoto, Japan) and mass spectrometer (Shimadzu, Kyoto, Japan). The temperature of the injector was set to 250 °C. High-purity helium was used as the carrier gas, with a column flow rate of 0.81 mL min^−1^ and a pre-column pressure of 73.0 kPa. After injection, the oven temperature was kept at 150 °C for 3.5 min, increased at 20 °C/min to 200 °C, held for 5 min, then increased at 5 °C/min to a final temperature of 280 °C, where it was held for 30 min. The sample injection volume was 1 μL with a split ratio of 50:1. The MS was operated in electron impact (EI) mode with an electron energy of 70 eV. Furthermore, the source temperature was set at 200 °C, and the transfer line temperature was 250 °C. The mass spectrometer was set to scan from *m*/*z* 50 to 600. There was a 3.5 min solvent delay time. Nonadecanoic acid (C19:0) (Sigma Aldrich, Dorset, UK) was used as an internal standard and its peak area was used to determine the amount of each fatty acid based on the areas of all peaks and the known concentration of the standard added.

## 5. Conclusions

In conclusion, the DHA production was optimized applying BBD under response surface methodology by fitting a second-order model to the response data. The optimized parameters for enhanced DHA accumulation by *Thraustochytrium* sp. ATCC 26185 was mostly close to the predicted values. Under cultivation using the determined optimal medium, the predicted value of DHA production yield was 1.77 g/L and the measured experimental value was 1.68 g/L in a shake-flask. Microbial DHA can be extracted and supplied to the food and pharmaceutical industries using this cultivation method, or the microbial biomass could be directly introduced as poultry feed and as a fish supplement in aquaculture.

## Figures and Tables

**Table 1:** Factors and experimental domain for the screening with Plackett-Burman design.

Factors	Associated Variable	Low Level (−1)	High Level (+1)
Glucose (g/L)	$X_{1}$	40	60
Yeast extract (g/L)	$X_{2}$	8	12
Sea salt (g/L)	$X_{3}$	20	30
Fermentation volume (mL)	$X_{4}$	200	300
Inoculum volume (%)	$X_{5}$	7	10
Temperature (°C)	$X_{6}$	25	30
Initial pH	$X_{7}$	6	8
Agitation (rpm)	$X_{8}$	150	200

**Table 2:** Plackett-Burman design for screening variables (in coded level) with DHA production as the response.

Runs	$X_{1}$	$X_{2}$	$X_{3}$	$X_{4}$	$X_{5}$	$X_{6}$	$X_{7}$	$X_{8}$	DHA (g/L)
1	1	1	−1	−1	−1	1	−1	1	0.890
2	1	−1	1	1	1	−1	−1	−1	0.263
3	1	1	1	−1	−1	−1	1	−1	0.494
4	−1	−1	−1	1	−1	1	1	−1	0.407
5	−1	−1	−1	−1	−1	−1	−1	−1	0.706
6	1	−1	−1	−1	1	−1	1	1	0.318
7	−1	1	1	−1	1	1	1	−1	0.382
8	−1	1	1	1	−1	−1	−1	1	0.742
9	−1	−1	1	−1	1	1	−1	1	0.722
10	−1	1	−1	1	1	−1	1	1	0.359
11	1	1	−1	1	1	1	−1	−1	0.401
12	1	−1	1	1	−1	1	1	1	0.363

**Table 3:** Experimental ranges and levels in the experimental design.

Factors	Range and Level		
	−1	0	+1
$X_{1}$: Fermentation volume (mL)	140	180	220
$X_{2}$: Inoculum volume (%)	4	5	6
$X_{3}$: Initial pH	6	6.5	7

**Table 4:** The Box-Behnken design matrix for coded variables along with actual and predicted responses.

Run	$X_{1}$	$X_{2}$	$X_{3}$	DHA (g/L)		
				Actual Response	Predicted Response	Residual
1	0	0	0	1.17	1.22	−0.058
2	−1	−1	0	1.54	1.48	0.068
3	0	0	0	1.14	1.22	−0.081
4	1	0	−1	1.22	1.16	0.057
5	−1	0	1	1.71	1.77	−0.057
6	0	0	0	1.22	1.22	5.800 × 10^−4^
7	0	1	−1	1.55	1.54	1.55
8	0	−1	1	1.57	1.58	−0.011
9	1	0	1	1.15	1.15	7.112 × 10^−4^
10	0	1	1	1.72	1.65	0.067
11	0	−1	−1	1.26	1.33	−0.067
12	0	0	0	1.22	1.22	−8.220 × 10^−3^
13	1	−1	0	1.16	1.15	9.994 × 10^−3^
14	−1	0	−1	1.39	1.39	−7.112 × 10^−4^
15	1	1	0	1.13	1.20	−0.068
16	−1	1	0	1.70	1.71	−9.994 × 10^−3^
17	0	0	0	1.37	1.22	0.15

**Table 5:** ANOVA results of quadratic model for DHA production (*R*^2^ = 0.9210; Pred *R*^2^ = 0.7290; Adj *R*^2^ = 0.8559; CV% = 6.58).

Source	SS	*df*	MS	*F*-Value	Prob > *F*	
Model	0.72	9	0.08	9.93	0.0031	significant
$X_{1}$	0.36	1	0.36	44.21	0.0003	
$X_{2}$	0.04	1	0.04	5	0.0604	
$X_{3}$	0.067	1	0.067	8.34	0.0234	
$X_{1}X_{2}$	8.46 × 10^−3^	1	8.46 × 10^−3^	1.05	0.34	
$X_{1}X_{3}$	0.039	1	0.039	4.88	0.063	
$X_{2}X_{3}$	5.08 × 10^−3^	1	5.08 × 10^−3^	0.63	0.4537	
$X_{1}^{2}$	5.05 × 10^−7^	1	5.05 × 10^−7^	6.25 × 10^−5^	0.9939	
$X_{2}^{2}$	0.11	1	0.11	13.07	0.0086	
$X_{3}^{2}$	0.086	1	0.086	10.7	0.0136	
Residual	0.057	7	8.08×10^−3^			
Lack of Fit	0.025	3	8.44 × 10^−3^	1.08	0.4516	not significant
Pure Error	0.031	4	7.80 × 10^−3^			
Cor Total	0.78	16				

SS, sum of squares; *df*, degrees of freedom; MS, mean square.

## Abbreviations

DHA	Docosahexaenoic acid
RSM	Response surface methodology
BBD	Box-Behnken Design
ANOVA	Analysis of variance
DO	Dissolved oxygen
